# Autophagy Modulation as a Treatment of Amyloid Diseases

**DOI:** 10.3390/molecules24183372

**Published:** 2019-09-16

**Authors:** Zoe Mputhia, Eugene Hone, Timir Tripathi, Tim Sargeant, Ralph Martins, Prashant Bharadwaj

**Affiliations:** 1 Centre of Excellence for Alzheimer’s Disease Research and Care, School of Medical and Health Sciences, Edith Cowan University, Nedlands, WA 6009, Australia; zoe.mputhia@gmail.com (Z.M.); e.hone@ecu.edu.au (E.H.); r.martins@ecu.edu.au (R.M.); 2 Department of Biochemistry, North-Eastern Hill University, Meghalaya 793022, India; timir.tripathi@gmail.com; 3 Hopwood Centre for Neurobiology, SAHMRI, Adelaide, SA 5000, Australia; tim.sargeant@sahmri.com; 4 School of Biomedical Science, Macquarie University, Sydney, NSW 2109, Australia; 5 School of Pharmacy and Biomedical Sciences, Curtin Health and Innovation Research Institute (CHIRI), Faculty of Health Sciences, Curtin University, Bentley, WA 6102, Australia

**Keywords:** amyloid, autophagy, clearance, toxicity, lysosome, Alzheimer’s disease, Parkinson’s disease, polyglutamine, Tau protein, beta amyloid, α-synuclein, Huntington’s disease

## Abstract

Amyloids are fibrous proteins aggregated into toxic forms that are implicated in several chronic disorders. More than 30 diseases show deposition of fibrous amyloid proteins associated with cell loss and degeneration in the affected tissues. Evidence demonstrates that amyloid diseases result from protein aggregation or impaired amyloid clearance, but the connection between amyloid accumulation and tissue degeneration is not clear. Common examples of amyloid diseases are Alzheimer’s disease (AD), Parkinson’s disease (PD) and tauopathies, which are the most common forms of neurodegenerative diseases, as well as polyglutamine disorders and certain peripheral metabolic diseases. In these diseases, increased accumulation of toxic amyloid proteins is suspected to be one of the main causative factors in the disease pathogenesis. It is therefore important to more clearly understand how these toxic amyloid proteins accumulate as this will aide in the development of more effective preventive and therapeutic strategies. Protein homeostasis, or proteostasis, is maintained by multiple cellular pathways—including protein synthesis, quality control, and clearance—which are collectively responsible for preventing protein misfolding or aggregation. Modulating protein degradation is a very complex but attractive treatment strategy used to remove amyloid and improve cell survival. This review will focus on autophagy, an important clearance pathway of amyloid proteins, and strategies for using it as a potential therapeutic target for amyloid diseases. The physiological role of autophagy in cells, pathways for its modulation, its connection with apoptosis, cell models and caveats in developing autophagy as a treatment and as a biomarker is discussed.

## 1. Introduction

Amyloids are aggregates of proteins that become folded into a structure that allows multiple copies of that protein to accumulate, forming potentially toxic fibrous deposits in cells and tissues [1]. While other amyloid proteins have the common role in structural support or motility, amyloids in humans are most commonly associated with the pathology observed in various chronic diseases [2]. In these pathological amyloids, the fibrous proteins aggregate into toxic forms that causes cell death, which leads to tissue and organ damage, culminating into clinical symptoms [3]. Some cells, such as neurons, are more vulnerable to protein aggregation—which is evident when observing neurodegenerative disorders. One of the most widely known amyloid protein is the amyloid beta (Aβ) protein, which is known to play a critical role in the pathogenesis of AD [4]. Aβ was discovered in 1984, as the major component found in neuritic plaques in the cerebral parenchyma of AD brains [5]. Later, Aβ was found to be a normal physiological product of the cleavage of its parent protein amyloid precursor protein (APP) [5]. Years after this discovery, amyloid association in the pathogenesis of PD, polyglutamine disorders such as Huntington’s disease (HD) and spinocerebellar ataxia (SCA) type 3 (otherwise referred to as Machado-Joseph disease), Creutzfedlt-Jakob disease, diabetes type 2, motor neuron diseases, amyotrophic lateral sclerosis (ALS) and peripheral tissue diseases such as familial amyloid polyneuropathy (FAP) [3] have been identified, in which all of the diseases involved are progressive disorders and are associated to high mortality and morbidity [2]. While the association between pathology and amyloid is widely known, the primary function of many amyloid proteins, including Aβ, is still unknown and remains controversial with experimental procedures, suggesting that it may have normal biological functions [6].

Cellular degradation and clearance pathways in the body assist in clearing misfolded proteins and preventing amyloid aggregation, therefore preventing the pathogenesis of amyloids in the body [7]. It is the dysfunction of these pathways that have shown to result in the accumulation of amyloid proteins like Aβ in the AD brain [7]. Protein aggregation is dynamic, and growing evidence shows that it is the small oligomer species that are the main toxic species. This is evident in studies showing high stability and resistance of the oligomeric intermediates of Aβ to degradation and clearance [8]. More so, Aβ oligomers have been demonstrated to even alter proteasomal clearance [8]. Despite protein aggregation or amyloidogenesis being hypothesized to be the underlying factor that causes various amyloid diseases, the connection between protein aggregation and tissue degeneration is not completely understood [9]. Recent studies have focused on a particular cellular degradation pathway known as autophagy where its dysfunction is implicated in AD and other amyloid diseases and its modulation may provide useful therapeutic pathways in treatment, which will be covered in this review. This pathway is responsible for degradation and recycling of misfolded or aggregated proteins and damaged organelles as well as other cellular components [10]. Other amyloid clearance pathways will be discussed in this review as well.

## 2. Clearance Pathways for Amyloid Proteins

To prevent unstable proteins from misfolding or aggregating, the protein folding process is tightly regulated through a network of cellular proteostasis pathways that ensures misfolded proteins do not accumulate [11]. Proteostasis is maintained in healthy cells through balance of the biological pathways, including protein synthesis, degradation and protein quality control (PQC) [12]. This complex proteostasis network coordinates these biological systems with intra- and extracellular molecular chaperones [13] and their regulators. Some of these chaperones, especially lens α-crystallins and milk caseins, operate in an ATP-independent manner to ensure that proteins are stabilized and do not misfold and aggregate into amyloid fibrils [11]. Bearing in mind that approximately 30% of proteins that are newly synthesized are misfolded, the influx of misfolded proteins calls for continuous operation of the PQC system [12]. The PQC system uses three main approaches to ensure that the misfolded or aggregated proteins do not threaten the survival of the cell and proteostasis is maintained. These include refolding, degradation and recycling, where the proteins are sequestered as inclusion bodies within the cell—all of which involve the function of molecular chaperones [14]. The degradation system involves two major systems: ubiquitin-proteasome system (UPS), which involves ubiquitin tagging of the target proteins followed by degradation by the proteasome [15], and the autophagy system, which is discussed later in this review. The proteostasis network has been shown to decline with age due to facing a range of both internal and external stresses that increase with age [16]. Studies implicate a dysfunction of this system to the abnormal protein assembly and amyloidogenesis in neurodegenerative diseases [14]. Increasing proteostasis network capacity therefore represents a promising pharmacological target that could delay the onset of age-related diseases [16].

Accumulation of the Aβ peptide into oligomers and amyloid plaques depends on the rate at which the Aβ peptide is synthesised and cleared [17]. There are several mechanisms that can achieve the clearance of Aβ peptide [17]. Examples of non-enzymatic clearance pathways include Aβ drainage in interstitial fluid drainage pathway and transport of amyloids into the circulation across walls of blood vessels [17]. Enzymatic pathways for Aβ degradation include insulin-degrading enzyme (IDE), neprilysin, matrix metalloproteins (MMPs), plasmin, angiotensin converting enzyme (ACE), and endothelin-converting enzyme (ECE) [17,18]. Notably, enzymes IDE, ACE and NEP have shown decreased activity levels in the hippocampus of AD patients (a region associated with Aβ deposition) which may suggest possible therapeutic strategies for AD [18]. Additionally, some of the enzymes above have even been successfully evaluated in animal models; however, their exact roles in regulating Aβ levels requires further investigation [18].

Another neurotoxic amyloid protein is α-synuclein, which is the pathological hallmark of PD, multiple system atrophy, and dementia with Lewy bodies. It is abundant in the presynaptic terminals that are responsible for the release of neurotransmitters [19]. Thus, despite its precise function remaining unknown, it appears that the main function of α-synuclein is to control the release of neurotransmitters, as well as dopamine regulation [20]. In PD, α-synuclein is the major fibrillar component of Lewy bodies, which consist of cytosolic protein inclusions [21]. These Lewy bodies spread and accumulate in dopaminergic neurons found in the substantia nigra of the brain, causing neuronal cell death [21]. Both the major degradation pathways, UPS and autophagy pathways are responsible for α-synuclein clearance [12]. Similar to AD and PD, HD is characterised by aggregation of the polyglutamine expanded huntingtin (polyQ-Htt) protein [22], while ALS is characterised by mostly mutant TAR DNA-binding protein 43 (TDP-43) aggregation as well as superoxide dismutase 1 (SOD1) aggregation, although shared pathogeny between these ALS-linked proteins is still uncertain [12]. Similar to Aβ, Tau protein and a-synuclein, both UPS and autophagy pathways are involved in the clearance of TDP-43, SOD-1 and polyQ-Htt [12].

## 3. Autophagy Pathway

It has been shown that one of the fundamental causes for the accumulation and deposition of toxic proteins is the inadequate clearance by autophagy, which is a part of the wider lysosomal system [23]. This pathway is a crucial degradation pathway for unwanted or damaged organelles and proteins [24]. Dysfunction of the autophagy-lysosomal system has been implicated in the pathogenesis of many neurodegenerative diseases that feature increased accumulation of amyloid protein deposits. At the same time, stimulating this pathway to clear the toxic amyloid proteins has gained interest as a therapeutic strategy [25].

The autophagic process involves a series of steps that are responsible for delivering vestigial intracellular macromolecules, damaged organelles and other cellular debris into the cell’s lysosome for degradation [24]. Distinguished by their manner of delivery into the lysosomes, three autophagy subtypes have been identified: micro-autophagy, chaperone-mediated autophagy and macro-autophagy [26]. Macroautophagy is the most common form of autophagy to be discussed in this review, and is herein referred to as autophagy. This autophagy mechanism is used in eukaryotes when long-living proteins and organelles need to be catabolized [24]. The autophagy process begins when an isolation membrane (phagophore) is formed and this step is known as the nucleation process [26]. The phagophore elongates and fuses along the edges, enclosing the cellular debris to form an autophagosome. The autophagosome either fuses with the lysosome directly to form an autolysosome where the contents are digested and the inner membrane is degraded by lysosomal hydrolases or initially fuses with late endosomes to form amphisomes which later fuse with lysosomes [26,27].

### 3.1. Autophagosome Generation

**Figure 1:**
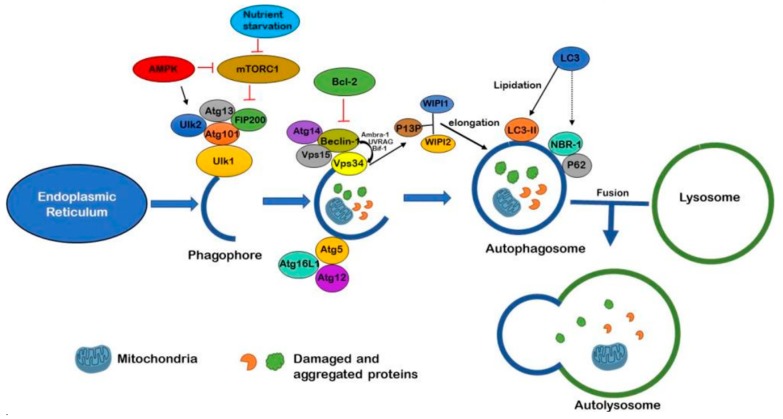
Regulation of autophagy mediated degradation: Autophagosome formation begins when phagophore is produced from ER initiated by either AMPK activation or mTOR inhibition by nutrient starvation. Once autophagosome formation is initiated, the ULK1 complex (composed of Atg13, Ulk2, Atg101 and Fip200) is phosphorylated. Vps4 complex is positively regulated and the developing phagophore elongates. Beclin-1 together with Ambra-1, Bif-1 and UVRAG activate Vps34, generates P13P—which then facilitates Atg16L1-Atg5-Atg12 recruitment. Beclin-1 mediated autophagosome generation can be inhibited by Bcl-2. P13P binds with WIPI1 and WIP2 and promotes phagophore elongation. LC3-I is lipidated to form LC3-II. LC3 additionally anchors adaptor proteins p62 and NBR-1 onto the phagophore for substrate targeting, and phagophore closes to become an autophagosome. Fusion of autophagosome and lysosome create the autolysosome, which digests the substrates and recycles back into the cytoplasm.

As illustrated in Figure 1, formation of the autophagosome begins via mechanistic target of rapamycin (mTOR) inhibition or 5′ adenosine monophosphate-activated protein kinase (AMPK) activation. The mTOR, a protein kinase specific to serine/threonine, is a crucial part of two core complexes: mTOR complex 1 (mTORC1) and mTOR complex 2 (mTORC2) [28]. The mTORC1 complex comprises of the mTOR regulatory-associated protein and is the primary initiator of autophagy. The phagophore first begins when a piece of membrane buds off of the endoplasmic reticulum (ER). An enzyme known as Unc-51-like kinase 1 (ULK1) is phosphorylated at its activation site by AMPK and this catalyses the phosphorylation of other components as it is accompanied by the Atg1-Ulk1 complex, which consists of ULK1, ULK2, FIP200, Atg13 and Atg101 [29]. This positively regulates the Vps34 complex activity—which is composed of Atg14, Vps34, Vps15 and Beclin-1—through phosphorylation [30]. This regulation allows for the complex to transfer from the cytoskeleton of the cell to the developing phagophore [31]. Beclin-1 in this complex interacts with Ambra-1, Bif-1 and UVRAG to activate Vsp34. Vps34 activity further generates phosphatidylinositol 3-phosphate (P13P) which binds with its effectors known as WIPI1 and WIPI2. This catalyses specific reactions that allow for elongation of the phagophore. The provision of P13P to the phagophore facilitates in the recruitment of the Atg16L1-Atg5-Atg12 complex which assists in targeting particular proteins or substrates to the developing phagophore [32]. When this Atg complex has bound to the phagophore, Atg7 and Atg3 allows for microtubule-associated proteins 1A/1B light chain 3B (LC3) to be lipidated into LC3-II, allowing for elongation of the phagophore and engulfing of cytoplasmic debris, leading to the complete autophagosome formation. LC3 enables p62 and NBR-1 to attach to autophagosome as well, which allows for selectivity of substrate to the pathway [30].

### 3.2. Substrate Targeting

It was previously suspected that autophagy was a non-selective process, but recent studies have shown that there are various forms of autophagy—in particular chaperone-mediated autophagy, mito- and macroautophagy—which target particular substrate subgroups [31]. Recently identified autophagic receptors have been found to respond to important cellular events that cause damage, such as pathogen invasion, organelle injury or aggregated proteins [31]. These receptors, as well as other selectivity adaptor proteins, bind the targeted substrates to fundamental autophagic components such as Atg5, Atg12, Atg16L, and LC3, which target the elimination of the substrate [33,34]. Specific autophagic receptors namely: NIX, p62, NBR1, HDAC6, OPTN and NDP52, recognise and facilitate elimination of ubiquitinated proteins, pathogens and peroxisomes as well [35]. PD-related proteins, Parkin (aka PARK2) and PINK1 are responsible for targeting damaged mitochondria for selective autophagy [36]. This is accomplished when PINK1—a serine-threonine kinase on the outer membrane of the mitochondria—is activated when mitochondrial function is compromised and the membrane is depolarised [36]. This further activates and recruits the cytoplasmic E3 ubiquitin ligase Parkin to the dysfunctional mitochondrion, where it binds and ubiquitinates the outer membrane proteins of the mitochondria [36]. Autophagy receptors p62, HDAC6 and LC3 are thereby recruited and, subsequently, selective autophagy is triggered [36].

### 3.3. Autophagosome-Lysosome Fusion

In this step, fusion occurs between the outer lipid bilayer membrane of autophagosomes and the lipid bilayer membrane of lysosomes. Soluble N-ethylmaleimide sensitive fusion attachment receptor proteins (SNARE) are important for lipid bilayer fusion and are therefore essential for autophagosome-lysosome fusion [37]. Other proteins involved in the fusion are Rab family proteins, Rubicon (beclin-1-binding protein), as well as the Phosphoinositide 3-kinase (PI3K) complex [37]. Autophagosome-lysosomal fusion opportunities are increased when cells direct the autophagosome to travel along the microtubules towards the perinuclear region of the microtubule-organizing centre (MTOC) of the neuron where lysosomes exist in abundance [38]. The dynein-dynactin complex mediates this movement of the autophagosomes to the perinuclear region [39] and loss of dynein causes immense build-up of autophagosomes within the neuronal process [40]. Additionally, the kinesin-dependent plus-end directed transport is important for the correct positioning of autophagosomes, as kinesin KIF5B depletion inhibits autophagy and further results in clustering of autophagosomes in a perinuclear manner [39]. During stress, lysosomes move towards the MTOC to fuse with autophagosomes, increasing the rate of fusion [41]. At this stage, the neurons are specifically vulnerable to disruption due to the vast amount of autophagosomes in the nerve terminals and axons that depend on retrograde transport to complete digestion of the cellular debris [31]. Movement of autophagosomes can be inhibited when injected at a microscopic level with antibodies against LC3 [39]. This further indicates the role of LC3, which, together with Rab7, forms an adaptor complex with the novel FYVE and coiled-coil domain that contains protein FYCO1 [39]. This complex promotes the microtubule plus end-directed transport of autophagosomes [42]. Throughout the retrograde journey, most autophagosomes will first fuse with the late endosomes to form amphisomes [38]. Amphisome formation requires some late endosome formation proteins including Rab11, HOPS (homotypic fusion and protein sorting) complex and ESCRT (endosomal sorting complexes required for transport) complex [31].

### 3.4. Lysosomal Digestion

The final stage of autophagy is the digestion of the autophagic contents in the lumen of the lysosome as well as the release of metabolites required for signalling and reuse [43]. Lysosomes are involved in several significant physiological processes including plasma membrane repair, cholesterol homeostasis, cell signalling and cell death, which makes it central to cellular homeostasis [44]. Lysosomal components include integral lysosomal membrane proteins (LMPs) and soluble lysosomal hydrolases (commonly referred to as acid hydrolases) [45]. Two of the most abundant LMPs are lysosome-associated membrane protein 1 (LAMP1) and LAMP2, which are responsible for protecting the lysosomal membranes from digesting themselves [45]. Acid hydrolases (e.g., nucleases, proteases, glycosidases, phospholipases, sulfatases, phosphatases and lipases) are activated when the lumen of the autolysosome becomes acidified to a pH of 4.5 to 5 by vacuolar ATPase (V-type ATPase). The v-ATPase is a large protein complex that acts as a proton pump across the lysosomal membrane [46]. Upon acidification, hydrolases then carry out their function as degradative enzymes that break down the cell debris in the lysosome [47]. The resulting degraded macromolecules are then released and reused in the cell cytosol [48]. It is important to note that while lysosomes are important for clearance of macromolecules during autophagy, they significantly influence the early stages of autophagy [31]. This occurs via a vATPase-anchored signalling complex that reversibly docks mTORC1 for activation. This complex phosphorylates and inhibits the nuclear translocation of transcription factors such as transcription factor EB (TFEB) [49]. Upon translocation into the nucleus, TFEB upregulates the expression of many genes that are involved in the biogenesis of the lysosome and other genes needed for autophagosome formation [49].

### 3.5. Atg5/Atg7-Independent Autophagy

Molecules Atg5 and Atg7 are essential for autophagy induction. However, it has been observed that cells lacking Atg5 or Atg7 can still form autophagosomes/autolysosomes and achieve autophagic protein degradation when specific types of stressors are applied [48]. While lipidation of LC3I to LC3II is regarded to be a good indicator for autophagy, it does not occur during Atg5/Atg7-independent autophagy [48]. This process was regulated as well by various autophagic proteins including Ulk2 and Beclin-1 [48]. In contrast to conventional macroautophagy, the autophagosomes seemed to be formed in a Rab9-dependent manner by fusion of the developing autophagosome with vesicles obtained from late endosomes and trans-Golgi network [48]. This pathway, similar to conventional autophagy, can be inhibited by autophagy inhibitor 3-Methyl adenine (3MA) [50]. This indicates that autophagy can occur through at least two distinct pathways; Atg5/Atg7-dependent conventional and -independent alternative pathway.

### 3.6. Non-Canonical Autophagy Pathway

In non-canonical autophagy, the formation of the autophagosome does not require the entire set of the Atg proteins or Beclin-1 and has no response to autophagy inhibitor 3MA [51]. Knockdown of Beclin-1 or its binding partner Vps34 does therefore not block non-canonical autophagy. For this reason, it is sometimes referred to as the Beclin-1 independent pathway [51]. However, this pathway differs from the Atg5/Atg7 independent pathway as it requires activity of Atg7 for lipidation of LC3I [52].

## 4. Role in Amyloid Diseases

Autophagy dysfunction can occur in the different stages in the autophagic pathway and lead to various diseases. Impairment in the formation of autophagosome has been observed in chronic neurodegenerative disease such as HD, AD, and PD [53]. PD is characterised by α-synuclein-containing inclusions and studies demonstrate a disruption of multiple stages of autophagy—in particular during the formation of the omegasome (autophagosome precursor) [54]. Atg9, Rab1A and α-synuclein are normal regulators of omegasome formation. In PD, Rab1A is inhibited by the increased levels of α-synuclein concentrations which causes a mis-localisation of the autophagy protein Atg9 and therefore a decrease in omegasome formation [54]. In HD, autophagy impairment is observed at the stage of autophagosome membrane recognition of substrates for degradation [55]. Autophagosomes are formed properly, although the balance between protein synthesis and degradation is altered in HD [55]. It is suggested that autophagosomes in the brain samples of HD patients have unusual lack of substrate cargo. It is suggested that Htt aggregates bind to Beclin-1 leading to Beclin-1 depletion, which then causes interference in the nucleation process and eventually in cargo recognition [55,56].

Disruption of cargo recognition for sequestration can also occur due to mutations in the p62-encoding SQSTM1 gene, which are associated with sporadic ALS, frontotemporal dementia (FTD), and Paget’s disease of bone marrow and disorders featuring p62-positive intra-neural inclusion [57]. Mutant p62 can disrupt ubiquitinated and aggregated protein clearance by autophagy [57]. Mutations in vasolin containing protein/p97 causes disruptions in selective autophagy and lead to FTD and Paget’s disease as well as inclusion-body myopathy by accumulation of immature autophagosomes containing ubiquitin-positive substrates [58]. Mutant PINK1 and Parkin encoding genes hinder mitophagy, thus causing accumulation of damaged mitochondria with possible initiation of apoptosis. These mutant genes account for most autosomal-recessive cases of PD [59]. Loss of function of LAMP2 type 2a (LAMP2A), inhibits the substrates of chaperone mediated autophagy to the lysosome [60]. In fact, loss of autophagic capacity through severe homozygous or compound heterozygous mutations in LAMP2 leads to Danon’s disease. This loss of function occurs when LAMP2A binds with misfolded and aggregated proteins, [60].

Studies have shown that mutations in dynein-dynactin complex lead to axonal Charcot-Marie-Tooth type 2 (CMT2) and lower motor neuron disease [61]. Additionally, dysregulation of this complex may be fundamental to spinal and bulbar muscular atrophy (Kennedy disease) [61]. Dynactin mutations have led to a decrease in the dynein complex activity in the motor neurons of ALS patients as well [61]. Furthermore, these mutations lead to accumulation of both p62-positive inclusions and autolysosomes as well [61]. A study was recently conducted with mice with D251E mutation in the VPS33A protein, a tethering protein and core subunit of the HOPS complex, which functions in autophagosome-lysosome fusion [62]. The results proposed a dual role of the VPS33A(D251E) mutation in increasing assembly of HOPS complex and associating with the SNARE complex, impairing the lysosome formation that leads to Purkinje cell loss in these mice [62].

Lysosomal storage disorders (LSDs)—most of which are characterized by severe neurodegenerative phenotypes—illustrate the close link between lysosomal dysfunction and neurodegeneration. In most LSDs, defects in various different lysosomal enzymes leads to deficits in autophagic turnover of macromolecules such as proteins, glycolipids and mucopolysaccharides, causing severe autophagic pathology in the brain [63]. One example of a severe neurological LSD that is caused by loss of function mutations in lysosomal enzyme cathepsin D is neuronal ceroid lipofuscinosis (Batten disease). This severe neurodegenerative disorder is associated with early onset neurological deterioration and death in childhood [64]. Similar phenotypes have been observed in mice after deletion of cathepsin D [64]. Microtubule-mediated transport of autophagosomes in the axon is susceptible to disruption when the microtubule binding tau protein is hyperphosphorylated [65]. This disruption prevents autophagosome-lysosome fusion, leading to autophagosome accumulation within the dystrophic neurites [65]. In fact, disruption of lysosomal proteolysis observed in AD shows a striking resemblance to autophagy neuropathology in LSDs and is more marked compared to any other late-onset brain disease [66].

Mutations in Presenilin-1 (PS1) protein causes familial AD. PS1 is necessary for lysosome acidification and therefore protease activation. PS1 mutations disrupt the lysosomal functions and significantly increases neuropathological severity and onset of familial AD [67]. This is evident in studies of transgenic APP/PS1 mutant AD model mice, which show elevated levels of LC3-II and autophagic vesicles (AVs) accumulation in the neurites [68]. In late-onset AD, the strongest genetic risk factor is the protein variant ApoE4 that is encoded by APOE allele which codes for a cholesterol transport protein [69]. ApoE4 destabilizes the lysosome membranes in an allele-specific way. Lysosomal proteolysis is disrupted as well when Rab5 is abnormally upregulated, thus speeding up the endocytotic process and causing protein and lipid cargoes to accumulate in late endosomes [70]. Mutant APP, APP duplication, high levels of dietary cholesterol and ApoE4 are AD risk factors that promote Rab5 upregulation as well [71]. Lysosomal dysfunction caused by impeded proteolysis, membrane damage and disrupted integrity are all similarly affected by accumulated Aβ peptide, reactive oxygen species, oxidized lipoproteins and lipids as observed in AD [72,73].

Overall, as observed above, autophagic dysfunction occurs in several neurodegenerative diseases at various stages of the autophagic process, and contributes to aggregate formation—eventually causing neuronal cell death. Autophagy dysfunction in the early stages of the autophagy process—during substrate sequestration and autophagosome formation—are observed in PD, HD, and Lafora’s disease. Dysfunction during substrate recognition and selective autophagy occurs in PD, ALS, Paget’s disease and SCA type 3. Dysfunction of autophagosome-lysosome fusion can occur as well in ALS, CMT2, frontotemporal dementia, and Kennedy’s disease. Whereas, dysfunction in lysosomal digestion occurs predominantly in AD and PD.

## 5. Activation of Autophagy as a Therapeutic Target for Amyloid Diseases

Studying the connection between autophagy and neurodegenerative diseases has now brought about the interesting question as to whether autophagy modulation could reduce protein aggregation and cell degeneration. Inhibition of the mTOR pathway has received the most attention in the development of drugs that stimulate autophagy in amyloid diseases. Autophagy is induced when mTORC1 is phosphorylated, leading to inhibiting the main autophagy complex that consists of Atg13, FIP200 and ULK1. Rapamycin is a widely used autophagy activator that binds with immunophilin FK506-binding protein (FKBP12), forming a complex that inhibits the mTORC1 kinase activity [74]. A study recently revealed that elevation of mTOR activity occurs in the hippocampus and neocortex of 3×TG-AD mice [52]. The study also showed that inhibiting mTOR signalling with rapamycin in a pharmacological manner reduced Aβ and Tau protein pathology and significantly rescued cognitive impairment [75].

The mTOR pathway not only modulates autophagy, but has a function in several other fundamental cell processes in growth and metabolism as well [76]. To prevent potential harmful side-effects that may occur with pharmacological mTOR inhibition, more studies are now focusing on mTOR-independent pathways that modulate autophagy. Several pathways have been discovered, mainly: inositol, Ca^2+^/caplain, cAMP/Epac/Ins (1,4,5)P3 and JNK1/Beclin-1/P13KC3 pathways [77]. A novel small molecule known as GTM-1 has been shown to diminish Aβ neurotoxicity via mTOR independent autophagy [78]. Another way autophagy is activated is by inhibiting activation of mTOR through AMPK pathway (Figure 1) [79]. Small molecules such as tyrosine kinase inhibitor Nilotinib have been found to induce autophagy via AMPK pathway activation by using an mTOR-independent pathway [80]. Additionally, anti-histamine Latrepirdine (dimebon) has been found to enhance autophagy in both yeast and mouse models through enhanced mTORC1 complex activity [81,82]. A disaccharide with pharmacological chaperone activity known as Trehalose has shown to reduce Aβ accumulation and assist in removal of abnormal proteins as well [83]. It possibly acts via AMPK activation and enhances clearance of aggregated huntingtin, α-synuclein and tau protein while promoting cytoprotective effects in cell and transgenic mouse models [77]. In APP/PS1 mice, it has shown to reduce deposits of Aβ—thereby rescuing their learning impairment [84].

Although autophagy activation is a promising intervention, there are some caveats to consider. While rapamycin is a potent autophagy activator, it has immunosuppressive effects as well as other pleiotropic effects resulting from the inhibition of mTOR, which also controls important cell processes including translation, cell growth and metabolism [77]. In cell models with impaired lysosomal clearance, inducing autophagy has shown to accelerate pathology, suggesting that the success of any autophagy-based intervention may depend on whether lysosomal clearance is functional. Augmenting autophagic function earlier in disease course before lysosomal deficits appear will therefore be a limiting factor for autophagy-based therapies [31].

One major drawback is the translatability of AD drugs from animal models to human clinical trials [85]. Additionally, AD pathology in humans develops over decades, while in transgenic mice the disease develops in just a few months and only with familial AD mutations [85]. Another discrepancy between most AD mouse models and humans is the lack of neurofibrillary tangles—which is an important hallmark of AD. Although tau protein hyperphosphorylation occurs in these mice, no neurofibrillary tangles develop and therefore these transgenic mice only model certain aspects of disease [85]. Additionally, effects of autophagy activation may vary significantly depending on the physiological state of the cell—especially during proteotoxic stress. With regards to testing autophagy modulators in animal models, there is no congruent evidence to demonstrate that autophagy activation can provide benefits in late stage disease with pre-existing pathology. In fact, some studies suggest that autophagy activation may be harmful in ageing conditions with pre-existing pathology [86].

## 6. Interplay of Autophagy and Apoptosis

Programmed cell death or apoptosis shares a number of biochemical pathways with autophagy and together they regulate stress response and cell survival [87]. Apoptosis is distinguished by a chain of morphological events, including nuclear condensation and fragmentation and blebbing of the plasma membrane, causing apoptotic-body formation [87]. Biochemical changes that accompany apoptosis include the effector caspases 3, 6 and 7, mitochondrial outer membrane permeabilization and catabolic hydrolase activation, which is responsible for macromolecule degradation of the cell, including DNA [87]. Autophagy sequentially antecedes apoptosis due to stimulation of autophagic response after stress is applied [87]. Once this stress surpasses an intensity threshold or a critical duration in which autophagy cannot acclimatize or endure, apoptotic and non-apoptotic lethal mechanisms are activated [87]. Autophagy can be inactivated if apoptosis is initiated in the cell, due to caspase-mediated cleavage of crucial autophagic proteins [87]. In general terms, autophagy could be directly linked to apoptosis by either autophagy controlling the likelihood of apoptosis or apoptosis controlling rate of autophagy [88].

Modulation of the interactions between autophagic Beclin-1 protein and the members of the Bcl-2 anti-apoptotic family of proteins, composed of Bcl-2, Bcl-Xl and Mcl-1, is one of the key mechanisms for autophagy regulation [89]. The interaction is controlled by a number of proteins that have the ability to either inhibit or enhance this Beclin-1/Bcl-2 interaction so as to activate or suppress autophagy, respectively [89]. While members of the Bcl-2 family are widely known for their anti-apoptotic properties, their roles as autophagy inhibitors is becoming more clear [89]. Additionally, their protective function is shown by their ability to antagonize their pro-apoptotic counterparts, Bax and Bak, and therefore preventing cell apoptosis [90]. Bcl-2 family members have been shown to react with autophagic protein Beclin-1, through the BH3 domain of Beclin 1. It is because of this that Beclin 1 is known as a Bcl-2 homology (BH-3) domain only protein [91]. Upon this interaction, Beclin-1 is prevented from assembling the phagophore and thereby inhibiting autophagy [90]. Bcl-2 therefore functions as an anti-apoptotic protein as well as an anti-autophagic protein through this interaction. Upon stress, Beclin-1 detaches from Bcl-2, thus Vps34 is activated and, subsequently, autophagy is induced [92]. Since autophagy modulation proposes a novel therapeutic direction for various common diseases, modifying the Beclin-1/Bcl-2 interaction with chemical compounds is a promising therapeutic avenue. This was evident in a recent study conducted in mice generated with F121A mutation in Beclin-1 that decreased its interaction with Bcl-2 [93]. Upon this disruption in the interaction, higher levels of basal autophagic flux were observed as well as a significant increase in lifespan and health span of mutant F121A knock-in mice compared to their wild-type littermates [93]. These results proved the disruption to be not only an effective mechanism for increased autophagy, but also for premature ageing prevention, health span improvement and longevity promotion in mammals.

Autophagy has been considered as a survival mechanism against brain ischemia mediated neuronal apoptosis. Autophagy is activated following an ischemic insult, but it may exert dual roles in cell death or survival during these two processes [94]. To date, the dual roles of autophagy in ischemia have not been fully clarified. In 2005, Yan et al. first showed that myocardial ischemia in pigs triggered autophagy via the increased expression of LC3 and cathepsin B and D21 [95]. Importantly, they proposed that autophagy could serve as a homeostatic mechanism to inhibit apoptosis and to limit the deleterious effects of chronic ischemia. In 2008, Carloni et al. showed that activation of autophagic pathways is a possible protective mechanism in the early stage of the brain ischemia. Beclin-1 was significantly increased in neurons shortly after neonatal hypoxic-ischemic injury–both in the hippocampus and in the cerebral cortex [96]. In contrast to the protective effects of autophagy, several reports indicate that ischemia mediated autophagy may be a process leading to cell demise. In 2006, Adhami et al. showed that in adult mice with hypoxia many damaged neurons exhibited features of autophagic and/or lysosomal cell death with the activation of proapoptosis AIF/caspase signaling pathways [97]. In 2007, Rami et al. reported a dramatic elevation in Beclin-1 and LC3 levels in rats challenged by cerebral ischemia [98]. A subpopulation of Beclin 1-upregulated cells expressed the active form of caspase-3 as well, implying that enhanced autophagy may be a process leading to cell demise. In 2008, Koike et al. showed increased autophagosome formation and extensive death of hippocampal neurons in neonatal mice subjected to hypoxic-ischemic injury [99]. A deficiency in the autophagy gene Atg7 provided nearly complete protection from neuronal death, indicating that Atg7 is a critical upstream gene involved in multiple neuronal death pathways. In addition, in a rodent permanent focal ischemia model, inhibition of autophagy reduced ischemic injury and autophagy activation, suggesting that the autophagy-lysosomal pathway is involved in the neuronal death induced by focal ischemia [100]. More recently, Ulamek-Koziol et al. showed that rats demonstrated dysregulation of expression of Beclin-1, BNIP 3 (BCL2 Interacting Protein 3), and caspase 3 genes in medial temporal lobe cortex to transient brain ischemia [101,102]. It was found that autophagy gene Beclin-1 was not significantly modified at all time points after ischemia, whereas mitophagy and caspase 3 genes were upregulated. It is suggested that mitophagy process may accompany apoptosis during brain ischemia [101,102].

Autophagy shows tumor suppressive qualities as well, as dysfunctional autophagy has been implicated in tumor development. Studies even propose an inverse biological link between AD and cancer [103]. In fact, there is growing interest in targeting Beclin-1/Bcl-2 interaction for identification of autophagy and apoptosis activators for reducing tumor growth. BH3 domains in pro-apoptotic proteins (known as BH3-only proteins) are structural templates for the design of BH3-mimetic molecule with the ability to inhibit the anti-apoptotic role of Bcl-2 protein family in cancer cells, thus triggering apoptosis of tumor cells [93]. In cancer, apoptotic mechanisms are disrupted whereas in contrast, AD is associated with increased neuronal death caused by Aβ and tau protein deposition. It is important to note that because autophagy is generally considered protective against apoptosis. Also, it has been identified as a mechanism for tumor cell survival and resistance towards chemotherapeutic treatment [104]. Additionally, most anti-cancer drugs, as well as ionizing radiation, increase autophagy in tumor cells. For this reason, autophagy inhibition appears to be potential mechanism to improve the response to drug treatment in cancer [104]. Similar to amyloid diseases, there is considerable obscurity in developing approaches to manipulate autophagy in cancer and it appears to be grossly dependent on the physiological state of the organism. In summary, multiple apoptotic signalling pathways may act together in a coordinated manner and form a complex network to regulate autophagy activation. Thus, the relationship among the signalling pathways involved in autophagy activation as well as the different roles of autophagy during amyloid accumulation and apoptosis remains a challenging research topic for future investigation.

## 7. Cell Models for Clearance of Protein Aggregates

Stimulating autophagy mediated clearance of protein aggregates is an important therapeutic target in protein misfolding disorders featuring amyloid protein accumulation in affected cells and tissues. A variety of cell models expressing wild type or pathogenic isoforms of the amyloid proteins have been used for investigating intracellular protein degradation mechanisms and drugs that promote the clearance of amyloid proteins such as α-synuclein, Tau protein, Aβ and proteins containing glutamine repeats (polyQ) such as Htt for developing novel treatment strategies. Activation of protein degradation systems has been proposed to be a potential strategy for removing amyloid proteins, but it still remains unclear how effectively protein aggregates can be degraded by these systems to promote cell survival.

Aggregation and toxicity of a-synuclein in neuronal cells is central to PD pathogenesis. The A53T mutation in the SNCA (Synuclein Alpha) gene encoding for α-synuclein—resulting in increased formation of Lewy body inclusion bodies with aggregated α-synuclein in the brain—is associated with PD. To assess α-synuclein clearance in cells, a recent study expressed the pathogenic A53T mutant of α-synuclein in human embryonic kidney cells (HEK293) cells. In HEK293 cells, Rab7 over-expression reduced α-synuclein accumulation. Clearance of α-synuclein is associated with increased levels of Rab7 containing vesicles—presumably representing autolysosomes. Rab7 over-expression reduced apoptosis as well [105].

Similarly, for Tau protein, findings from Guo et al. study in 2016 established a cell model using a HEK293-derived QBI-293 cell line with inducible expression of mutant Tau protein, to study the turnover of its aggregates [106]. Tau protein aggregates in cells expressing full-length mutant Tau protein were gradually cleared when production is suppressed. This clearance was partially mediated by the autophagy-lysosome pathway. Importantly, residual Tau protein aggregates left after the clearance phase led to a re-occurrence of Tau protein pathology once expression was turned on again.

Clearance of polyQ proteins has been investigated in human central nervous system (CNS) derived neuroblastoma cells, SK-N-SH expressing the N-terminal fragment of TATA-binding domain protein (TBP) enclosing various polyQ repeats tagged with enhanced green fluorescence protein (EGFP). In this work, a triazole derivative, OC-13, was found to accelerate autophagic clearance of aggregates of the EGFP-conjugated chimeric protein that enclosed 79 polyQ repeats (Q79-EGFP) [107]. Clearance of polyQ has been investigated in neuronal cells stably expressing or transiently expressing wild type or mutant ARpolyQ. Dynein inhibition correlated with a reduced accumulation and an increased clearance of mutant ARpolyQ, SOD1, truncated TARDBP/TDP-43 and expanded polyGP C9ORF72 products. Misfolded protein clearance was mediated by the proteasome and correlated with the upregulation of the HSPA8 cochaperone BAG1. Overexpression of BAG1 increased the proteasome-mediated clearance of these misfolded proteins as well [108]. Studies have used a tetracycline regulatable design to distinguish newly formed versus preformed inclusions as well, and have shown that two proteins previously implicated in the autophagic clearance of expanded polyglutamine inclusions, HspB7 and Alfy, affect distinct cellular processes to affect aggregate burden [109]. Similarly, inducible cell models have been used to investigate a new pathway for maintaining protein homeostasis mediated by the proteasome shuttle factor UBQLN2 (Ubiquilin 2). The 26S proteasome degraded polyubiquitylated substrates by recognizing them through ubiquitin receptors, but substrates are delivered by reversibly bound shuttles as well. This process was active in the cell nucleus, where aggregate clearance by autophagy does not act. Mutations in UBQLN2 were defective in chaperone binding, impairing aggregate clearance, and causing cognitive deficits in mice [110].

A number of studies have investigated the aggregation, neurotoxicity and metabolism of APP and its cleavage products, including pathogenic proteins APP- Carboxy Terminal Fragments (CTFs including C99 and C83) and Aβ; however, there are several critical gaps in our understanding of its intracellular clearance mechanisms. Studies have used neuronal cells stably expressing APP to measure CTFs and Aβ in the secreted media [111]. HEK293 cells stably transfected with human APP695 (APPHEK293) and N2a cells stably transfected with human APP695 harboring the Swedish double mutation (SwAPP-N2a). Resveratrol and related molecules have been identified to inhibit Aβ accumulation in cell lines as well. Two of these molecules, RSVA314 and RSVA405 were found to facilitate Calcium/calmodulin-dependent protein kinase kinase 2 (CaMKKbeta)-dependent activation of AMPK, to inhibit mTOR, and to promote autophagy to increase Aβ degradation by the lysosomal system [112]. Although activating autophagy by mTOR inhibition has shown benefits in AD, studies caution the stimulation of this pathway during ageing and in the presence of pre-existing protein aggregation and impairment in autophagy [113].

**Figure 2:**
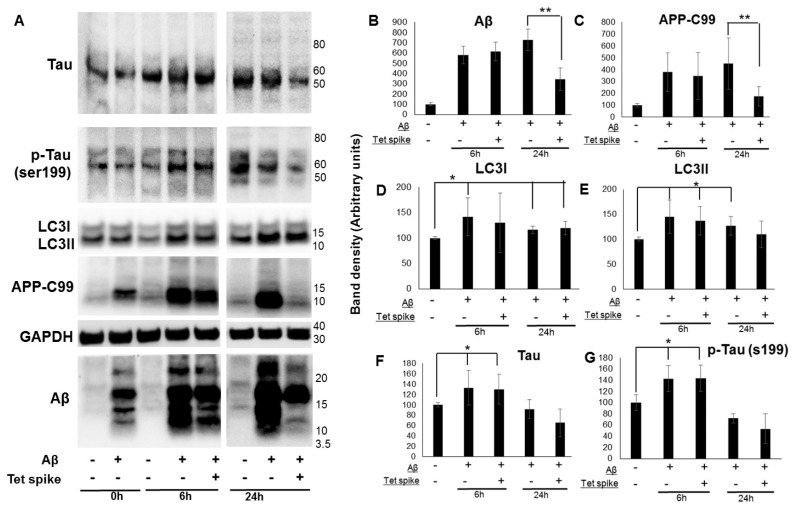
Clearance of APP-C99 and Aβ in an AD neuronal cell model. Clearance of APP-C99 and Aβ was assessed in MC65, a human derived cell line that produces Aβ from a stably transfected precursor protein APP fragment C99. MC65 cells were grown without tetracycline for two days to activate APP-C99 and Aβ production, followed by spiking with tetracycline to block APP-C99/Aβ production. After spiking with tetracycline (1 µg/mL), cell lysates were collected at 6 and 24 h to measure levels of Aβ (**B**), APP-C99 (**C**), LC3I (**D**) LC3II (**E**), Tau protein (**F**), and phosphorylated Tau protein (ser199) (**G**) by western immunoblotting analysis. Representative image of the Aβ clearance assay is shown here (**A**). APP-C99 and Aβ levels were ~50% lower at 24 h in cells spiked with tetracycline compared to tetracycline depleted cells (** *p* < 0.001, n = 4), suggesting clearance. Aβ producing MC65 cells (tetracycline depleted) showed increased accumulation of Tau protein, phosphorylated Tau protein (ser199) at 6 h and autophagy markers LC3I and LC3II (* *p* < 0.005, n = 4) at both 6 and 24 h, suggesting increased autophagosome synthesis and autophagy activation.

Here, we used a human CNS derived cell line that produces Aβ from a stably transfected precursor protein APP-C99 to study clearance of Aβ, Tau protein and changes in autophagic markers (Figure 2). We adapted this cell model to monitor Aβ clearance using “Tet spiking” to suppress Aβ and APP-C99 production. MC65 cells were grown without tetracycline for two days to induce APP-C99/Aβ production, followed by spiking with tetracycline to block C99/Aβ production. After spiking with tetracycline, cell lysates were collected at 6 and 24 h to measure levels of Aβ, APP-C99, LC3, Tau protein, and phosphorylated Tau (ser199) protein (Figure 2). APP-C99 and Aβ levels were ~50% lower at 24 h in cells spiked with tetracycline compared to tetracycline depleted cells (Figure 2B,C). It was notable that certain Aβ oligomer species were more resistant to clearance at 24 h. A decrease in Tau and phosphorylated Tau (ser199) protein was also observed at 24 h in spiked cells (Figure 2F,G), but the effect was not significant. Aβ producing MC65 cells (tetracycline depleted) showed increased accumulation of Tau protein, phosphorylated Tau (ser199) protein at 6 h (Figure 2F,G) and autophagy markers LC3I and LC3II at both 6 and 24 h (Figure 2D,E). Our findings showed increased accumulation of LC3 in cells producing APP-C99 and Aβ, which is notably similar to pathology observed in post-mortem AD brains—indicating disruption in the lysosomal clearance of autophagosomes [66]. Using this cell-based assay, we observe as well that neuronal cells can measurably eliminate Aβ protein aggregates and not all aggregates appear to be equally available for degradation. This new assay can therefore not only determine at what step a modifier might influence aggregate burden, but can be used to provide new insights into how protein aggregates are targeted for degradation as well.

## 8. Autophagy as a Biomarker for Amyloid Diseases

Dysfunction in the autophagy-lysosome pathway is an early, conspicuous feature in neurological amyloid diseases. Because this process is highly dysfunctional in neurodegenerative diseases, and, importantly, because these diseases are genetically linked with the lysosomal system [114], autophagic and lysosomal markers in accessible biofluids may be useful for predicting disease and in response to interventions. As the lysosomal system is very dysfunctional in AD, it is no surprise that studies have reproducibly found lysosomal system components that are significantly altered in CSF and blood components. Across AD, PD, and primary tauopathies, these altered lysosomal system markers include autophagic proteins such as LC3B, lysosomal hydrolases such as CTSD, and lysosomal membrane proteins such as LAMP2 (Table 1).

In AD, changes in early endocytosis and autophagy can be detected in CSF [115]. Armstrong and colleagues found increased amounts of early endosomal protein EEA1, and the GTPases RAB3 and RAB7. Interestingly, in this study, the robust AD-risk factor gene product—PICALM—was not altered in the same CSF samples. The autophagic cargo protein LC3 was increased in abundance as well. In contrast, autophagic proteins ATG5 and ATG6 were not.

More work has focused on lysosomal proteins in AD—perhaps as a consequence of the large amount of work performed on hydrolases in AD by Professor Nixon in the 1990s [31]. CTSD, an important lysosomal protease (discussed above), accumulates in and around amyloid plaques [116] and is robustly increased in biofluids as well. This was discovered in CSF, where, although an abundance of CTSD was increased—its specific activity was decreased, showing secretion of an inactive (likely immature) form [117]. CTSD was increased in brain-derived exosomes present in blood as well [118]. This study showed a remarkable increase in CTSD in AD patient blood compared with blood from controls. Other lysosomal enzymes are altered in blood in AD as well. The lysosomal glycosidases β-galactosidase and β-hexosaminidase are increased in plasma during AD. Conversely, these same enzymes were deficient inside peripheral blood mononuclear cells. α-mannosidase was decreased in peripheral blood mononuclear cells as well [119].

It is not just AD where lysosomal system changes could provide useful biomarkers. Lysosomal system changes are observed in PD in CSF and blood derived cells as well. Similar to AD, PD is genetically associated with the lysosomal system [120], and displays lysosomal system pathology as well [114], albeit in different ways to AD; whereas overall lysosomal system markers appear to increase in AD—they decrease in PD. Autophagic markers LC3B and ATG5 decrease in CSF in PD [121]. Multiple studies show lysosomal components appear to follow suit. In CSF, lysosomal membrane proteins LAMP1 and LAMP2 are decreased in PD compared with controls [121,122,123]. Lysosomal hydrolases are reduced CSF in PD as well, and these include α-mannosidase, β-mannosidase, glucocerebrosidase, and β-hexosaminidase [124]. Of note, whereas early endosomal protein EEA1 is increased in abundance in AD CSF—it is unchanged in PD CSF [122], likely reflecting differences in how the lysosomal system interacts with each disease. One study has explored lysosomal system changes in blood in PD [125]. This study found glucocerebrosidase activity was decreased in monocytes (but not lymphocytes) compared with controls. Importantly, this difference in activity was maintained even when GBA1 mutants were excluded (GBA1 codes for glucocerebrosidase and is a genetic risk factor for PD).

As pathology for late onset neurodegenerative diseases begins decades before the onset of overt clinical symptoms [126], the challenge for groups investigating lysosomal system changes is to look into mid-life, where changes likely begin. Lysosomal system-based biomarkers for late-onset neurodegenerative diseases offer both a vista on dysfunctional cell biology, and an important opportunity for tailoring therapeutic interventions that improves lysosomal function. While changes in lysosomal system markers in CSF are a good measure for cellular dysfunction in the brain, changes in plasma and leukocytes for both AD and PD hints at genomic variation that fundamentally alters lysosomal system processes in the body. As such, future research should focus on looking for lysosomal changes that occur in mid-life—rather than late in disease.

## Figures and Tables

**Table 1:** Altered lysosomal markers in neurodegenerative disease.

Disease	Biomarker Type	Lysosomal System Dysfunction	Study
AD	CSF	Increased EEA1, LAMP1, LAMP2, LC3, RAB3, RAB7	Armstrong et al. [115]
AD	Blood-derived brain exosomes	Increased CTSD, LAMP1, ubiquitinylated proteins	Goetzl et al. [118]
FTD	Blood-derived brain exosomes	Increased CTSD	
AD	CSF	Increased CTSD	Schwagerl et al. [117]
AD	Plasma	Increased β-hexosaminidase, β-galactosidase activity	Tiribuzi et al. [119]
	Peripheral blood mononuclear cells	Decreased β-hexosaminidase, β-galactosidase activity	
PD	Monocytes	Reduced glucocerebrosidase activity	Atashrazm et al. [125]
PD	CSF	Reduced α-mannosidase, β-mannosidase, glucocerebrosidase, and β-hexosaminidase activity	Balducci et al. [124]
PD	CSF	Decreased LC3B, ATG5, LAMP2, Beclin1	Youn et al. [121]
PD	CSF	Decreased LAMP1, LAMP2	
PSP	CSF	Decreased EEA1	
CBD	CSF	Increased LAMP1, LAMP2, LC3	Boman et al. [122]
PD	CSF	Decreased LAMP2 (specifically in female LRRK2-mutation carrying patients)	Klaver et al. [123]
